# Prevalence and correlates of comprehensive HIV/AIDS knowledge among adolescent girls and young women aged 15–24 years in Malawi: evidence from the 2015–16 Malawi demographic and health survey

**DOI:** 10.1186/s12889-021-11564-4

**Published:** 2021-08-04

**Authors:** Chrispin Mandiwa, Bernadetta Namondwe, Mtondera Munthali

**Affiliations:** 1 Elizabeth Glaser Pediatric AIDS Foundation (EGPAF), P.O. Box 2543, Lilongwe, Malawi; 2 University of Malawi, Kamuzu College of Nursing, Lilongwe, Malawi; 3 Ministry of Health, Mzuzu Health Centre, Mzuzu, Malawi

**Keywords:** HIV/AIDS, Comprehensive knowledge, Young women, Malawi

## Abstract

**Background:**

HIV epidemic remains a major public health issue in Malawi especially among adolescent girls and young women (AGYW). Comprehensive HIV/AIDS knowledge (defined as correct knowledge of two major ways of preventing the sexual transmission of HIV and rejection of three misconceptions about HIV) is a key component of preventing new HIV infections among AGYW. Therefore, the aim of this study was to identify the correlates of comprehensive HIV/AIDS knowledge among AGYW in Malawi.

**Methods:**

The study was based on cross-sectional data from the 2015–2016 Malawi Demographic and Health Survey. It involved 10,422 AGYW aged 15–24 years. The outcome variable was comprehensive HIV/AIDS knowledge. Data were analysed using descriptive statistics, bivariate and multivariable logistic regression model. All the analyses were performed using complex sample analysis procedure of the Statistical Package for Social Sciences to account for complex survey design.

**Results:**

Approximately 42.2% of the study participants had comprehensive HIV/AIDS knowledge. Around 28% of the participants did not know that using condoms consistently can reduce the risk of HIV and 25% of the participants believed that mosquitoes could transmit HIV. Multivariable logistic regression model demonstrated that having higher education (AOR = 2.97, 95% CI: 2.35–3.75), belonging to richest households (AOR = 1.24, 95% CI: 1.05–1.45), being from central region (AOR = 1.65, 95% CI:1.43–1.89), southern region (AOR = 1.65, 95% CI: 1.43–1.90),listening to radio at least once a week (AOR = 1.27, 95% CI: 1.15–1.40) and ever tested for HIV (AOR = 1.88, 95% CI: 1.68–2.09) were significantly correlated with comprehensive HIV/AIDS knowledge.

**Conclusions:**

The findings indicate that comprehensive HIV/AIDS knowledge among AGYW in Malawi is low. Various social-demographic characteristics were significantly correlated with comprehensive HIV/AIDS knowledge in this study. These findings suggest that public health programmes designed to improve comprehensive HIV/AIDS knowledge in Malawi should focus on uneducated young women, those residing in northern region and from poor households. There is also a need to target AGYW who have never tested for HIV with voluntary counselling and testing services. This measure might both improve their comprehensive HIV/AIDS knowledge and awareness of their health status.

## Background

Even though the number of AIDS-related deaths has dropped by 55% in Malawi since 2010, HIV epidemic still remains a major public health challenge in Malawi especially among adolescent girls and young women (AGYW) aged 15 to 24 years [1]. Young women are generally considered to be the most vulnerable group with regard to HIV/AIDS compared to their male peers in Malawi [2]. In 2016, the prevalence of HIV among Malawian AGYW was estimated to be 4.9% compared to 1% among young men [3]. Similarly, in 2018, the incidence of HIV was also high among young women with 9900 new HIV infections compared to 4200 new HIV infections among young men [4]. This reflects gender disparities in risk of HIV acquisition between young women and men. Therefore, understanding the dynamics of HIV transmission among AGYW is key to designing appropriate HIV prevention strategies.

Majority of new HIV infections among AGYW are transmitted through unprotected heterosexual intercourse [5–9]. Several studies conducted in low- and middle-income countries (LMICs) have suggested that inaccurate knowledge about HIV transmission and prevention methods among AGYW is one of the major contributing factors to risky sexual behaviours that predisposes them to the danger of acquiring and spreading HIV [10–13]. Thus, having comprehensive HIV/AIDS knowledge (defined as correct knowledge of two major ways of preventing the sexual transmission of HIV and rejection of three misconceptions about HIV) is a necessary component of averting the spread of HIV among AGYW. Comprehensive knowledge of HIV/AIDS enables people to assess their own risk and facilitates adoption of safer sexual practices [14, 15]. Comprehensive knowledge of HIV/AIDS also helps to reduce stigma and discrimination towards people infected and affected by HIV [16, 17]. Furthermore, comprehensive knowledge of HIV/AIDS helps individuals living with HIV to adhere to antiretroviral treatment [18–20]. Therefore, the importance of comprehensive HIV/AIDS knowledge in preventing HIV transmission cannot be overemphasized.

Although comprehensive HIV/AIDS knowledge plays a significant role in preventing the spread of HIV, there is paucity of evidence regarding magnitude and correlates of comprehensive HIV/AIDS knowledge among AGYW who have been disproportionally affected by new HIV infections relative to their male counterparts in Malawi. Previous studies conducted in developing countries have found that despite AGYW ever heard of HIV/AIDS, their comprehensive HIV/AIDS knowledge was low [21, 22]. It is thus crucially important to identify the correlates of comprehensive HIV/AIDS knowledge among AGYW. A better understanding of the correlates of comprehensive HIV/AIDS knowledge among AGYW can help policy makers and planners for HIV programmes to design appropriate HIV prevention strategies for reducing new HIV infections among AGYW in Malawi, which in turn may contribute towards achieving the Joint United Nations Programme on HIV/AIDS (UNAIDS) goal of ending the HIV/ AIDS epidemic by 2030. Therefore, the objective of this study was to identify the correlates of comprehensive HIV/AIDS knowledge among AGYW in Malawi.

## Methods

### Data source and study design

This study utilised data from the 2015–2016 Malawi Demographic and Health Survey (MDHS), a cross–sectional nationally representative population–based household survey. Study participants were selected using a two-stage stratified cluster sampling procedure. The first stage involved selection of the primary sampling units (clusters); the second stage involved the selection of households through systematic random sampling. All men aged 15–54 years and women aged 15–49 years who were either permanent residents or visitors to the household the night preceding the survey were eligible to be interviewed using the Men’s and Women’s Questionnaires, respectively. The data analysed in this study were collected using women’s questionnaire. A total of 24,562 women were successfully interviewed, yielding a response rate of 98%. We restricted our analyses to data for 10,422 AGYW aged 15–24 years. Further information regarding the sample selection procedure used in the 2015–2016 MDHS can be found in the publicly available survey report [3].

### Measurement of variables

#### Dependent variable

The dependent variable in this study was comprehensive HIV/AIDS knowledge. Participants were asked a set of five questions to evaluate their HIV/AIDS knowledge. Comprehensive HIV/AIDS knowledge was assessed by correctly knowing two major ways of preventing the sexual transmission of HIV: (1) consistent use of condoms during sexual intercourse and (2) having just one uninfected faithful partner help to reduce the chances of getting HIV. Furthermore, the assessment was based on rejecting the three most common local misconceptions about transmission or prevention of HIV: (3) a person can get HIV from a mosquito bite, (4) a person can get HIV by sharing food with a person who has HIV, and (5) a healthy-looking person cannot have HIV infection. Participants who responded correctly to all the five questions were considered to have comprehensive HIV/AIDS knowledge and were coded “1” while participants who responded with at least one incorrect and don’t know responses were regarded not to have comprehensive HIV/AIDS knowledge and were coded “0”.

#### Covariates

The covariates were selected based on a thorough literature review and their availability in the DHS data set [23–26]. The covariates included were: age (in two categories: 15–19 and 20–24), area of residence (rural vs. urban), region of residence (northern, central, southern), marital status (categorised as never married, currently married and formerly married), education level (no education, primary education and secondary or higher education), ever been tested for HIV(yes/no), religion(Christian, Muslim and no religion) and frequency of listening to radio (not at all, less than once a week and at least once a week). We considered this variable (frequency of listening to radio) other than frequency of watching TV and reading newspaper because there are a lot of HIV/AIDS programmes that are aired on different radio stations in Malawi. So, we wanted to assess if these programmes are making any positive impact (i.e. improving HIV/AIDS knowledge among AGYW in Malawi). Household wealth index was also considered as an independent variable. The wealth index is a composite measure of a household’s cumulative living standard and was constructed by the MDHS team using the principal component analysis (PCA) method by weighting each household assets (e.g. radios, refrigerators, televisions), housing characteristics (e.g. materials used for constructing the house), and access to basic services (e.g. source of drinking water) [27, 28]. After analysis, each household was assigned a wealth asset score, and based on that; the households were divided into five quintiles (poorest, poorer, middle, richer, richest).

#### Data analysis

Prior to analyses, all variables were assessed for accuracy, completion and credible values. Descriptive statistics were used to summarize the characteristics of the study sample and the results were presented as weighted frequencies and percentages. Bivariate analyses using chi-square test were performed to determine the distribution of the independent variables according to comprehensive HIV/AIDS knowledge of the participants (Yes/No). Variables that were significant at *p*-value of ≤0.25 during chi-square analysis were retained for logistic regression analysis [29]. Bivariate and multivariable logistic regression analyses were performed to identify correlates of comprehensive HIV/AIDS knowledge. Both crude and adjusted odds ratios together with their corresponding 95% confidence intervals (95% CI) were computed. Weighting, stratification and clustering variables created by the DHS were used throughout the analysis. All the analyses were performed using Complex Sample Analysis procedure of the Statistical Package for Social Sciences (SPSS version 22) to account for complex survey design. A *p-*value of less than 0.05 was considered statistically significant.

#### Ethics statement

The 2015–2016 Malawi Demographic and Health Survey was approved by Malawi Health Sciences Research Committee and the Institutional Review Board of ICF Macro in Calverton Maryland, USA. Consent for participation into the survey was obtained from all the respondents by enumerators on behalf of the National Statistical Office of Malawi and the DHS programme. A written request was submitted to the DHS programme and permission was granted to use the data for this study.

## Results

### Characteristics of respondents

Table 1 displays the socio-demographic characteristics of the 10,422 participants who were included in the analysis. Slightly more than half of the respondents (50.5%) were aged 15–19 years and nearly two thirds of the participants (64.7%) had primary education. About 46.9% of the participants were currently married and 45.4% of the participants were from southern region. Majority of the participants (81.8%) were rural inhabitants and 22.7% were from richest households. Regarding religion, the majority (86.8%) of the participants were Christians. Around 53.3% of the participants did not listen to radio at all and 69.5% of the respondents had ever been tested for HIV.

**Table 1:** Socio-demographic characteristics of study participants

Variables	Frequency(n)	Percent (%)
**Age**		
15–19	5263	50.5
20–24	5159	49.5
**Education**		
No education	454	4.4
Primary level	6740	64.7
Secondary level or higher	3227	31.0
**Marital status**		
Never married	4828	46.3
Currently married	4888	46.9
Formerly married	706	6.8
**Region**		
Northern	1159	11.1
Central	4536	43.5
Southern	4726	45.4
**Residence**		
Rural	8530	81.8
Urban	1892	18.2
**Household wealth status**		
Poorest	2084	20.0
Poorer	2117	20.3
Middle	1945	18.7
Richer	1908	18.3
Richest	2368	22.7
**Religion**		
Christians	9044	86.8
Muslim	1340	12.9
No religion	36	0.3
**Frequency of listening to radio**		
Not at all	5551	53.3
Less than once a week	1873	18.0
At least once a week	2998	28.8
**Ever tested for HIV**		
No	3174	30.5
Yes	7248	69.5
**Total (n)**	10,422	100.0

### Comprehensive knowledge of HIV/AIDS

**Fig. 1:**
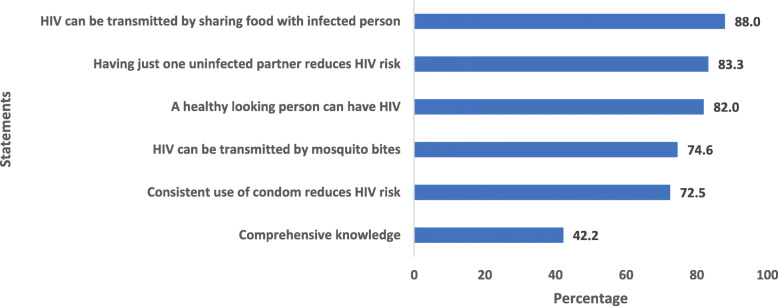
Percentage of young women who responded correctly to questions assessing knowledge about HIV/AIDS in Malawi

Figure 1 shows the percentage of correct answers about knowledge of HIV/AIDS among young women in Malawi. About 88% of the respondents were aware that HIV cannot be transmitted by sharing food with someone who has HIV. Around 83.3% of the participants knew that having one uninfected faithful partner can reduce the chances of getting HIV, 82.0% of the participants knew that a healthy-looking person can have HIV, 74.6% of the respondents knew that HIV cannot be transmitted by mosquito bites and 72.5% of respondents answered correctly that consistent condom use can reduce the risk of HIV. Overall, 42.2% of young women in Malawi had comprehensive knowledge of HIV/AIDS.

### Socio-demographic variables correlated with comprehensive HIV/AIDS knowledge among young women in Malawi

Table 2 shows the results of bivariate analysis between the covariates and comprehensive HIV/AIDS knowledge. The following variables: age, education, region, area of residence, household wealth index, religion, frequency of listening to radio and ever tested for HIV were significantly correlated with comprehensive HIV/AIDS knowledge (*p* < 0.05).

**Table 2:** Socio-demographic variables correlated with comprehensive HIV/AIDS knowledge among young women in Malawi

	Comprehensive HIV/AIDS Knowledge					
**Independent variables**	**No**		**Yes**			
	**n**	**%**	**n**	**%**	**Chi-Square**	***P-Value***
**Age**						< 0.001
15–19	3145	59.8	2118	40.2	17.00	
20–24	2876	55.7	2283	44.3		
**Education**						< 0.001
No education	335	73.8	119	26.2	348.41	
Primary level	4243	63.0	2497	37.0		
Secondary level or higher	1442	44.7	1785	55.3		
**Marital status**						0.241
Never married	2747	56.9	2081	43.1	2.84	
Currently married	2859	58.5	2029	41.5		
Formerly married	415	58.8	291	41.2		
**Region**						< 0.001
Northern	751	64.8	408	35.2	27.09	
Central	2600	57.3	1936	42.7		
Southern	2669	56.5	2057	43.5		
**Residence**					39.12	< 0.001
Rural	5050	59.2	3480	40.8		
Urban	971	51.3	921	48.7		
**Household wealth status**						< 0.001
Poorest	1345	64.5	739	35.5	123.68	
Poorer	1307	61.7	810	38.3		
Middle	1157	59.5	788	40.5		
Richer	1025	53.7	883	46.3		
Richest	1187	50.1	1181	49.9		
**Religion**						0.002
Christians	5177	57.2	3867	42.8	12.05	
Muslim	815	60.8	525	39.2		
No religion	28	77.8	8	22.2		
**Frequency of listening to radio**						< 0.001
Not at all	3447	62.1	2104	37.9	94.53	
Less than once a week	1021	54.5	852	45.5		
At least once a week	1553	51.8	1445	48.2		
**Ever tested for HIV**						< 0.001
No	2114	66.6	1060	33.4	145.40	
Yes	3907	53.9	3341	46.1		
**Total (n)**	6021	57.8	4401	42.2		

### Logistic regression of comprehensive HIV/AIDS knowledge among young women in Malawi

Table 3 shows the crude odds ratios (COR) and adjusted odds ratios (AOR) for the correlates of comprehensive HIV/AIDS knowledge among young women in Malawi. After adjusting for other variables in the multivariable logistic regression model, women who had primary education (AOR = 1.61, 95% CI: 1.30–2.01) and secondary or higher education (AOR = 2.97, 95% CI: 2.35–3.75) were more likely to have comprehensive knowledge about HIV/AIDS than those with no education. Similarly, women from central region (AOR = 1.65, 95% CI:1.43–1.89) and southern region (AOR = 1.65, 95% CI: 1.43–1.90) were more likely to have comprehensive knowledge about HIV/AIDS compared to women from northern region. We also observed that women from richer households (AOR = 1.29, 95% CI:1.13–1.48) and richest households (AOR = 1.24, 95% CI:1.05–1.45) were more likely to have comprehensive knowledge about HIV/AIDS compared to women from poorest households. Furthermore, women who were listening to radio less than once a week (AOR = 1.22, 95% CI:1.09–1.36) and at least once a week (AOR = 1.27,95% CI: 1.15–1.40) were more likely to have comprehensive knowledge about HIV/AIDS compared to women who were not listening to radio at all. Women who ever tested for HIV (AOR = 1.88, 95% CI:1.68–2.09) were more likely to have comprehensive knowledge about HIV/AIDS compared to women who had never tested. On the other hand, women who were married (AOR = 0.85, 95%CI: 0.76–0.95) were less likely to have comprehensive knowledge compared to their never married counterparts.

**Table 3:** Logistic regression of comprehensive HIV/AIDS knowledge among young women in Malawi (*n* = 10,422)

Variables	COR	95% CI	***P-value***	AOR	95% CI	***P-value***
**Age**						
15–19	1			1		
20–24	1.18	1.09–1.27	< 0.001	0.97	0.87–1.07	0.482
**Education**						
None	1			1		
Primary school	1.65	1.33–2.05	< 0.001	1.61	1.30–2.01	< 0.001
Secondary school or above	3.48	2.79–4.33	< 0.001	2.97	2.35–3.75	< 0.001
**Marital status**						
Never married	1			1		
Currently married	0.94	0.86–1.02	0.113	0.85	0.76–0.95	0.005
Formerly married	0.93	0.79–1.09	0.340	0.88	0.74–1.06	0.189
**Region**						
Northern	1			1		
Central	1.37	1.20–1.57	< 0.001	1.65	1.43–1.89	< 0.001
Southern	1.42	1.24–1.62	< 0.001	1.65	1.43–1.90	< 0.001
**Residence**						
Rural	1			1		
Urban	1.38	1.25–1.52	< 0.001	0.88	0.77–1.01	0.067
**Household wealth status**						
Poorest	1			1		
Poorer	1.13	0.99–1.28	0.062	1.08	0.95–1.23	0.225
Middle	1.24	1.09–1.41	0.001	1.14	0.99–1.30	0.056
Richer	1.57	1.38–1.78	< 0.001	1.29	1.13–1.48	< 0.001
Richest	1.81	1.61–2.04	< 0.001	1.24	1.05–1.45	0.011
**Religion**						
Christians	1			1		
Muslims	0.86	0.77–0.97	0.013	0.98	0.86–1.11	0.716
No religion	0.40	0.18–0.86	0.019	0.47	0.21–1.04	0.468
**Frequency of listening to radio**						
Not at all	1			1		
Less than once a week	1.37	1.23–1.52	< 0.001	1.22	1.09–1.36	< 0.001
At least once a week	1.52	1.39–1.67	< 0.001	1.27	1.15–1.40	< 0.001
**Ever tested for HIV**						
No	1			1		
Yes	1.71	1.56–1.86	< 0.001	1.88	1.68–2.09	< 0.001

Abbreviations: COR crude odds ratio, AOR adjusted odds ratio, CI confidence interval

## Discussion

The results indicate that comprehensive knowledge of HIV/AIDS among adolescent girls and young women in Malawi is low. We found that more than one quarter of young women in Malawi did not know that using condoms consistently can reduce the risk of HIV. Similarly, about one quarter of AGYW in Malawi believed that mosquitoes could transmit HIV. This is a worrisome finding as it suggests that some AGYW in Malawi are still lacking accurate knowledge of HIV transmission as well as prevention methods. This result is consistent with the findings of a study done in Ghana [30]. It is worth noting that knowledge alone is not sufficient to influence young women’s practices and behaviours regarding HIV prevention, other factors such as cultural and religious beliefs also influence individual’s practices and attitudes. Therefore, there is a need to consider cultural and other factors when designing HIV programmes in order to reduce the incidence of HIV among AGYW. The present study has revealed that education, region, household wealth status, frequency of listening to radio and ever been tested for HIV were significantly correlated with comprehensive HIV/AIDS knowledge.

This study has demonstrated a positive association between education and comprehensive HIV/AIDS knowledge. AGYW who had attained primary education and those who had secondary or higher education were more likely to have comprehensive knowledge of HIV/AIDS compared to those who had no education. This finding is consistent with the results from previous studies conducted in Kenya and other African countries [21, 22, 24, 25, 30]. There are various potential explanations for the positive correlation between increasing level of education and comprehensive HIV/AIDS knowledge. Women with higher education are likely to know how to read which makes them understand the content of written information such as newspaper articles easily compared to those without any formal schooling. Education also helps individuals to be proactive about their own health and to gather information to protect themselves against HIV. To reduce the risk of HIV spreading among AGYW, it is very crucial to reach out and provide correct HIV/AIDS information to AGYW with no education in communities.

The present study also found that region of residence was significantly correlated with comprehensive HIV/AIDS knowledge. Women residing in southern and central region were more likely to have comprehensive knowledge of HIV/AIDS compared to women residing in northern region. Our finding agrees with the results of studies conducted in Ethiopia and Uganda which also found variations in comprehensive knowledge of HIV/AIDS among women across the regions of these countries [31, 32]. In Malawi, the majority of tertiary education institutions which host many students, mainly young women, are based in central and southern regions other than the northern region. Therefore, young women in these two regions are likely to have comprehensive HIV/AIDS knowledge because they are more exposed to HIV information at the institutions than those who reside in the northern region. However, there is a need for further research to understand why young women from the north have lower chances of having comprehensive knowledge of HIV/AIDS compared to those from central and southern part of Malawi.

The current study found that married young women were less likely to have comprehensive HIV/AIDS knowledge compared to their never married counterparts. This finding is similar to the results of studies carried out in Ethiopia and Kenya [21, 22, 31]. Plausible explanation for this finding could be that most married young women enter into marriage at an early age before completing their studies. As a result, they may have poor understanding of HIV/AIDS related information. Thus, our findings suggest the need for tailor made HIV/AIDS education programmes for married young women to improve their comprehensive HIV/AIDS knowledge. Since this study has shown that voluntary counseling and testing (VCT) can improve comprehensive knowledge about HIV/AIDS, it is thus extremely important to target married young women with VCT services to improve their comprehensive HIV/AIDS knowledge and awareness of their health status.

In line with similar studies existing in the literature [24, 31], the findings of this study show that comprehensive knowledge of HIV/AIDS was significantly correlated with ever been tested for HIV. Young women who ever tested for HIV were more likely to have comprehensive knowledge about HIV/AIDS compared to those who were not tested for HIV. This could be due to the pre and post HIV test counselling sessions that health care providers offer to individuals seeking HIV testing service. The counselling sessions provide opportunity to clients to get important HIV/AIDS related information from health care providers. It is possible that these sessions help clients to enhance their HIV knowledge. To improve comprehensive knowledge of HIV/AIDS among young women, it is important to encourage young women to utilize HIV testing and counseling services. In addition, existing interventions to improve HIV/AIDS knowledge should be focused on young women who have never tested for HIV.

The study also revealed that AGYW belonging to richer and richest households as well as those who were listening to radio had comprehensive HIV/AIDS knowledge compared to their counterparts. These results are consistent with the findings of previous research conducted in Sub-Saharan African (SSA) countries, where most of the participants who had comprehensive knowledge about HIV/AIDS were listening to radio frequently and were from richest households [24, 32, 33]. A possible explanation for these findings is that AGYW belonging to richest households can easily afford and access information from media and other platforms compared to AGYW from poorest households. Taken together, these findings suggest a need to target women from poor households and those who do not listen to radio with appropriate interventions (such as HIV/AIDS awareness campaigns) that can increase their comprehensive knowledge about HIV/AIDS.

These results should be interpreted in the context of the following study limitations. Firstly, the data is confined to the last MDHS round (2015–2016) and current levels of comprehensive knowledge of HIV/AIDS among AGYW may have changed. Secondly, this study was based on secondary data analysis and we were unable to include other potential variables that might correlate with comprehensive HIV/AIDS knowledge but not available in the MDHS dataset. The study used data that were self-reported, which is prone to recall and social desirability bias. As a characteristic of all cross-sectional studies, this study was unable to conclusively establish temporal relationship between the covariates and outcome variable. Despite these limitations, this is the first study to explore the correlates of comprehensive HIV/AIDS knowledge among adolescent girls and young women aged 15–24 years in Malawi using a nationally representative sample. Therefore, the findings of this study would contribute towards developing innovative public health strategies to improve comprehensive knowledge of HIV/AIDS among young women in Malawi.

## Conclusions

The findings suggest that comprehensive HIV/AIDS knowledge among adolescent girls and young women in Malawi is low. Several variables including education, region of residence, household wealth status, frequency of listening to radio and ever been tested for HIV were significantly correlated with comprehensive HIV/AIDS knowledge among AGYW in this study. Lack of basic knowledge about HIV transmission as well as prevention methods among AGYW in Malawi might pose a challenge to efforts to halt the spread of the HIV epidemic. To reduce the incidence of HIV among AGYW, it is essential to design effective public health interventions that can increase comprehensive HIV/AIDS knowledge among AGYW in Malawi. Therefore, policy-makers and planners for HIV/AIDS programmes need to direct their efforts to AGYW with no formal education, those married, those residing in northern region and from poor households as well as those that do not listen to the radio at all. AGYW who have never been tested for HIV should be more intensely targeted with voluntary counselling and testing services to improve both their HIV/AIDS knowledge and awareness of their HIV status.

## Data Availability

The datasets used and analysed during the current study are available from the corresponding author on reasonable request. The datasets are also available at https://dhsprogram.com/. However, permission needs to be obtained from the Demographic Health Survey Programme Team.

## Abbreviations

AGYW: Adolescent girls and young women
AIDS: Acquired Immunodeficiency Syndrome
HIV: Human Immunodeficiency Virus
LMICs: Low- and Middle-Income Countries
MDHS: Malawi demographic health survey
MNHSRC: Malawi National Health Sciences Research Committee
SSA: Sub–Saharan Africa
